# The Role of Racial and Ethnic Factors in MicroRNA Expression and Risk for Type 2 Diabetes

**DOI:** 10.3389/fgene.2022.853633

**Published:** 2022-03-18

**Authors:** Elena Flowers, Alka M. Kanaya, Li Zhang, Bradley E. Aouizerat

**Affiliations:** ^1^ University of California, San Francisco, Department of Physiological Nursing, San Francisco, CA, United States; ^2^ University of California, San Francisco, Institute for Human Genetics, San Francisco, CA, United States; ^3^ University of California, San Francisco, Department of Epidemiology and Biostatistics, San Francisco, CA, United States; ^4^ University of California, San Francisco, Department of Medicine, Division of General Internal Medicine, San Francisco, CA, United States; ^5^ New York University Bluestone Center for Clinical Research, New York, NY, United States; ^6^ New York University Department of Oral and Maxillofacial Surgery, New York, NY, United States

**Keywords:** microRNA, diabetes, fasting blood glucose, biomarker, prediabetes, race/ethnicity, ancestry, diabetes prevention program

## Abstract

Prior studies focused on circulating microRNAs and the risk for complex diseases have shown inconsistent findings. The majority of studies focused on European and East Asian racial or ethnic groups, however, ancestry was not typically reported. We evaluated the risk for type 2 diabetes as an exemplar to show that race and ethnic group may contribute to inconsistent validation of previous findings of associations with microRNAs.

MicroRNAs (miRs) are emerging as “intermediary” biomarkers that capture the combined effect of gene-environment interactions. Circulating miRs found in serum and plasma are easily measured in blood and may have utility for determining risk for the development of complex diseases. More than 37 million people (11.3% of the population) in the United States have type 2 diabetes (T2D) and an additional 96 million (38% of the population) have prediabetes. (Centers for Disease Control and Prevention, 2022). However, there are marked differences in prevalence across racial and ethnic groups in the United States and globally. Given the complex etiology of T2D, both genetic (at times reflecting ancestry) risk factors as well as social determinants likely contribute to these observed differences. A number of prior studies identified associations between circulating miRs and risk for T2D. However, the findings from these studies are disparate. As with genetic association studies, one reason for these disparate findings includes demographic differences in the population sample (e.g., ancestral or genetic, racial or ethnicity). If expression of miRs is influenced by genetic ancestry as estimated by ancestry informative markers (AIMs), then defining a T2D miR “signature” may also reflect information about underlying genetic ancestry. In the absence of genetic information about a study sample, this signature could provide the opportunity to control for confounding based on differences in ancestry, discrete from differences in race or ethnicity, which would help parse out the separate impacts of genetic risk and social determinants (e.g., racism) on complex diseases when AIMs are not available. Given that miRs are responsive to environmental and behavioral factors, these biomarkers may also provide insights as to how ancestry impacts responses to social determinants across racial and ethnic groups. The purpose of this study was to test whether there were differences in associations between miRs and risk for T2D by racial or ethnic group that may reflect underlying confounding due to the impact of social determinants of health.

This study was a secondary analysis of data and biospecimens from participants in the Diabetes Prevention Program (DPP) trial that tested two approaches to prevention of T2D after 2-years. DPP sample characteristics, trial design, and methods have been described in detail previously. (The Diabetes Prevention Program, 1999; The Diabetes Prevention Program, 2000; The Diabetes Prevention Program, 2002). This analysis includes a randomly selected subset (*n* = 1,000) equally stratified by the intervention arm. We performed a literature review to identify miRs that were previously associated with prediabetes [100 mg/dl < fasting blood glucose (FBG) < 126 mg/dl)]. MicroRNAs are named in sequential order of entry into an annotation database, with the -3p or -5p suffix referring to which arm of the stem loop structure is represented (Ambros et al., 2003). From all miRs reported, we selected those that were statistically significant in at least two prior studies. Of those, miR-126-3p, miR-126-5p, miR-144, miR-192, and miR-23a were present in our existing dataset and were included in this study. The Multiplex Circulating MicroRNA Assay (Abcam, MA) was used to directly quantify miRs from plasma. Descriptive statistics were calculated to examine and evaluate the demographic and clinical characteristics of participants. (R, 2019) Expression of individual miRs was normalized using the set of miR probes (i.e., hsa-miR-92a-3p, hsa-mir-93-5p, hsa-miR-17-5p) identified by the geNorm algorithm for each experiment (Ambros et al., 2003). Spearman’s correlation coefficients were calculated to determine the associations between individual miRs and FBG (R, 2019). Subgroup tests were performed by sex, race, and ethnicity.

Sample characteristics are shown in Table 1. None of the miRs that we assessed were significantly associated with FBG in the study sample overall (Figure 1). However, when we stratified by sex, race, and ethnicity, we observed significant associations for miR-126-3p (*ρ* = 0.3, *p* < 0.05) in Hispanic men, miR-192 (*ρ* = 0.26, *p* < 0.05) in Black women, and miR-23a in males overall (*ρ* = −0.11, *p* < 0.05) and White males (*r* ^2^ = 0.16, *p* < 0.05).

**TABLE 1:** Demographic and clinical characteristics.

mean ± SD or n (%)	*n* = 1,000
Age	52 ± 10
Gender	
Male	322 (32.2)
Female	678 (67.8)
Bachelor’s degree or higher	489 (48.9)
Race and ethnicity^a^	
White	618 (61.8)
Black	193 (19.3)
Hispanic	146 (14.6)
Other	43 (4.3)
Body mass index (kg/m^2^)	33.7 ± 6.9
Fasting glucose (mg/dl)	107 ± 8
Fasting insulin (uU/ml)	26 ± 15
Triglycerides (mg/dl)	166 ± 98
Total cholesterol (mg/dl)	204 ± 35
HDL cholesterol (mg/dl)	46 ± 12
LDL cholesterol (mg/dl)	124 ± 32
Waist circumference (cm)	104 ± 15
Hip circumference (cm)	114 ± 15
Average systolic blood pressure (mm Hg)	124 ± 15
Average diastolic blood pressure (mm Hg)	78 ± 9

a Race and ethnicity were collected in the parent trial using these categories only with no disambiguation for race versus ethnicity.

**FIGURE 1:**
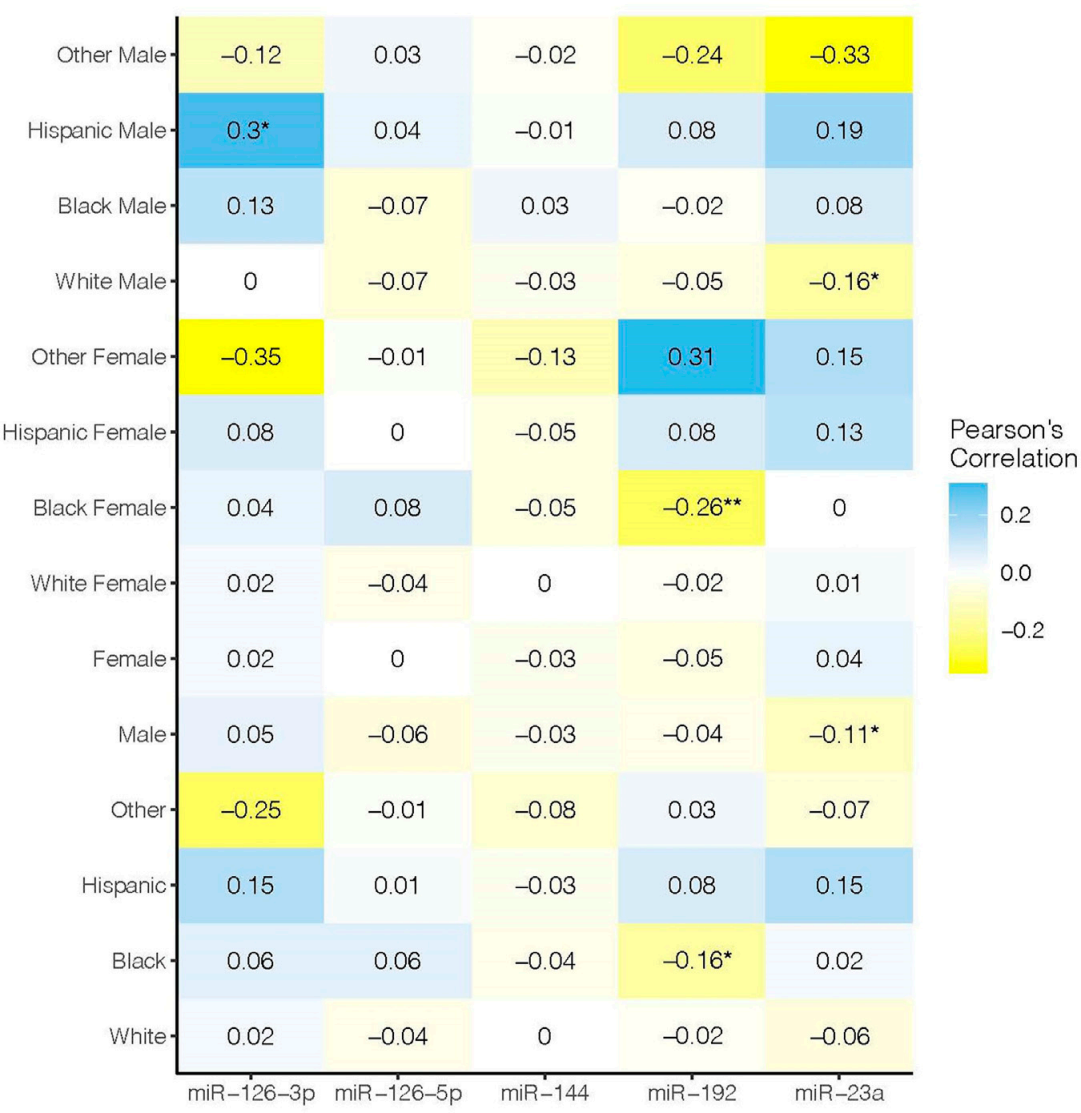
Rows show race/ethnic group. Columns show microRNAs assessed. Color intensity shows the magnitude of the correlation. Statistically significant values are denoted with an asterisk.

The scientific community is reaching a consensus that race and ethnicity should be operationalized as a social rather than biological construct (Flanagin et al., 2021). This is a critical step forward for conducting research focused on the impact of racism and other social determinants on health outcomes, distinct from the impact of genomic characteristics on health outcomes. While both categories of risk factors (i.e., social, genetic) are important for understanding the etiology of complex diseases like T2D, and which are often correlated, they should not be conflated. Because miRs are encoded from the genome, variability in the genetic sequence that is observed among ancestral groups may also be relevant to the genomic regions encoding for miRs, and therefore differences in expression and function of individual miRs. Prior studies focused on miRs have not typically applied robust approaches to account for this variability using AIMs. A major challenge to designing studies that control for ancestry is the need for genetic data. In contrast, one of the most promising features of circulating miRs is that they can be easily measured from blood collected in clinical settings and do not require nuclear genetic material. As has been used using protein-encoding genes, expression levels of a subset of miRs that reflects ancestral genetic influences. This “fingerprint” could then be used to control for underlying genetic differences between individuals that may impact observed associations between miRs and risk for complex traits, including T2D. This would augment the established ease of measuring miRs with methods to account for underlying genetic differences confounding observed statistical associations. While we did not validate prior findings on associations between individual miRs and risk for T2D, we showed that some of these associations were significant when we stratified by racial or ethnic group and sex. Prior studies have not consistently reported these characteristics, and no studies reported ancestral characteristics. As is well established in genetic association studies, differences in underlying genetic characteristics is a likely contributor to inconsistent validation of miRs across studies.

The strongest observed association was between miR-126-3p and FBG in Hispanic men. MiR-126 was one of the first miRs identified in a study focused on T2D, (Zampetaki et al., 2010), and numerous studies showed that this miR is likely related to endothelial function and complications from T2D and not the etiology of T2D itself (Zampetaki et al., 2010). Prior studies on T2D-related complications showed inconsistent relationships with vascular complications in men who identify as Hispanic (Haw et al., 2021). Our prior study did not provide evidence that miR-126 was significantly associated with prediabetes versus normal glucose tolerance in independent studies of United States Latin and Mexican origin participants (Flowers et al., 2021). Failure to adequately control for ancestry may contribute to these disparate findings, and there may be disproportionate risk in this group because of shared ethnic-based exposures that could be confounded by genetic (ancestral) differences between distinct groups of men who identify as Hispanic.

Similarly, we saw a moderate correlation between miR-192 and FBG in Black women. A recent review showed evidence that miR-192 is related to many diseases, including two studies of T2D, by targeting the forkhead box 01 (*FOX O 1*) gene to repress insulin resistance (Ren et al., 2021). One was performed on the European continent, although participants’ racial and ethnic characteristics were not described (Jaeger et al., 2018). The other was performed in the United States, and 83% of the sample self-reported as White with the rest of the sample categorized as Other (Nunez Lopez et al., 2019). Thus, we cannot determine how ancestry, race, or ethnicity are related to expression levels of miR-192 and risk for T2D.

This study also showed that miR-23a was associated with FBG in men overall and also White men. A prior study found associations between miR-23 in adipose tissue and insulin resistance (Lozano-Bartolomé et al., 2018). The study took place in Spain and approximately half the participants were reported as male. Several prior studies identified miR-23a associated with T2D-related retinopathy (Zhao et al., 2016; Santovito et al., 2021) and β-cell destruction in type 1 diabetes (Zhao et al., 2016; Grieco et al., 2017). Most of these studies were conducted on the European and Asian continents. Recent studies have shown that T2D is a heterogeneous condition, with several subtypes that are characterized by distinct clusters of characteristics (e.g., insulin resistance vs low insulin secretion). (Ahlqvist et al., 2018; Zou et al., 2019; Anjana et al., 2020; Herder et al., 2021). These subtypes feature varying risk for T2D-related complications like retinopathy (Grieco et al., 2017) and evidence for genetic differences between these subgroups of patients (Ahlqvist et al., 2018). Future studies that characterize genetic ancestry may help to inform whether there are genetic differences in miRs associated with T2D subgroups by ancestral origin.

We limited our assessment to the subset of miRs identified in at least two prior studies of prediabetes; additional miR are certain to be relevant. Historically, studies have often used self-reported race and ethnicity as a proxy for genetic ancestry. A more accurate etiological model would differentiate race and ethnicity as a social construct, discrete from genetic ancestry as a biological construct, when assessing for potential confounding (Borrell et al., 2021). The observed associations between miRs and FBG for some subgroups by race and ethnicity and sex in this study cannot parse the underlying risk (i.e., social determinants, genetic, both) without AIMS. For population-based studies of T2D that do not have AIM data, the establishment of a miR “fingerprint” that reflects underlying genetic differences would prove instrumental to miR studies that can estimate the contributions of social vs biological impacts on risk for T2D. Cohorts that possess AIM and miR data can be used to develop and validate such a miR fingerprint.

## Data Availability

The original contributions presented in the study are included in the article/Supplementary Material, further inquiries can be directed to the corresponding author.
